# Consequences of a high incidence of microsatellite instability and *BRAF‐*mutated tumors: A population‐based cohort of metastatic colorectal cancer patients

**DOI:** 10.1002/cam4.2205

**Published:** 2019-05-09

**Authors:** Kristine Ø. Aasebø, Anca Dragomir, Magnus Sundström, Artur Mezheyeuski, Per‐Henrik Edqvist, Geir Egil Eide, Fredrik Ponten, Per Pfeiffer, Bengt Glimelius, Halfdan Sorbye

**Affiliations:** ^1^ Department of Clinical Science University of Bergen Bergen Norway; ^2^ Department of Pathology Uppsala University Hospital Uppsala Sweden; ^3^ Department of Immunology, Genetics and Pathology Uppsala University Uppsala Sweden; ^4^ Department of Global Public Health and Primary Care, Lifestyle Epidemiology Group University of Bergen Bergen Norway; ^5^ Centre for Clinical Research Haukeland University Hospital Bergen Norway; ^6^ Science for Life Laboratory Uppsala University Uppsala Sweden; ^7^ Department of Oncology Odense University Hospital Odense Denmark; ^8^ Department of Oncology Haukeland University Hospital Bergen Norway

**Keywords:** colorectal neoplasm, microsatellite instability, proto‐oncogene proteins, B‐raf, prognosis, neoplasm metastasis, KRAS protein

## Abstract

**Background:**

Immunotherapy for patients with microsatellite‐instable (MSI‐H) tumors or *BRAF*‐inhibitors combination treatment for *BRAF‐*mutated (mut*BRAF)* tumors in metastatic colorectal cancer (mCRC) is promising, but the frequency of these molecular changes in trial patients are low. Unselected population‐based studies of these molecular changes are warranted.

**Methods:**

A population‐based cohort of 798 mCRC patients in Scandinavia was studied. Patient and molecular tumor characteristics, overall survival (OS) and progression‐free survival (PFS) were estimated.

**Results:**

Here, 40/583 (7%) tumor samples were MSI‐H and 120/591 (20%) were mut*BRAF*; 87% of MSI‐H tumors were mut*BRAF (non‐Lynch)*. Elderly (>75 years) had more often MSI‐H (10% vs 6%) and MSI‐H/mut*BRAF* (9% vs 4%) tumors. Response rate (5% vs 44%), PFS (4 vs 8 months), and OS (9 vs 18 months) after first‐line chemotherapy was all significantly lower in patients with MSI‐H compared to patients with microsatellite stable tumors. MSI‐H and mut*BRAF* were both independent poor prognostic predictors for OS (*P* = 0.049, *P* < 0.001) and PFS (*P* = 0.045, *P* = 0.005) after first‐line chemotherapy. Patients with MSI‐H tumors received less second‐line chemotherapy (15% vs 37%, *P* = 0.005).

**Conclusions:**

In unselected mCRC patients, MSI‐H and mut*BRAF* cases were more common than previously reported. Patients with MSI‐H tumors had worse survival, less benefit from chemotherapy, and they differed considerably from recent third‐line immunotherapy trial patients as they were older and most had mut*BRAF* tumor (non‐Lynch).

## 1 BACKGROUND

Colorectal cancer (CRC) is a heterogeneous group of tumors with a wide range of genetic changes. Microsatellite instability (MSI) is caused by mutations in DNA mismatch repair (MMR) genes, which leads to failure to repair errors that occur in DNA replication in repetitive sequences (microsatellites). This leads to accumulation of frameshift mutations in genes with microsatellites, also called MSI‐high (MSI‐H). Most MSI‐H tumors result from accumulated mutations during life but can also occur due to inherited MMR deficiency (Lynch syndrome). Most previous studies on metastatic CRC (mCRC) have reported that around 4% of the tumors are MSI‐H^1^, ^2^, ^3^, ^4^, ^5^ and 5%‐12% *BRAF* mutated (mut*BRAF*).^3^, ^4^, ^6^, ^7^ Most previous reports are based on patients included in clinical trials, and these patients are highly selected with both younger age and better performance status (PS) compared to patients in the general clinical practice.^8^, ^9^ Sporadic MSI‐H CRC is associated with a *BRAF* mutation in about 40%‐60% of the cases, whereas Lynch syndrome tumors are essentially *BRAF* wild‐type (wt*BRAF*).^10^, ^11^ In nonmetastatic CRC, MSI‐H is associated with less risk of recurrence and improved survival compared to microsatellite stable (MSS) tumors.^12^, ^13^ In mCRC, MSI‐H tumors appear to have poor prognosis, but the number of patients in these studies are limited.^2^, ^3^, ^4^, ^5^, ^6^, ^14^ However, mut*BRAF* has a strong negative prognostic impact in mCRC, but the possible relevance of MSI status for poor prognosis is not clarified.^3^, ^4^, ^5^, ^6^, ^15^, ^16^, ^17^

Recent studies have shown that mCRC patients with MSI‐H tumors respond to immunotherapy given mainly as third‐line treatment.^18^, ^19^, ^20^ The recently updated National Comprehensive Cancer Network guidelines recommend second‐line treatment with a PD‐1 inhibitor in patients with MSI‐H tumor and addition of BRAF‐inhibitors to standard treatment in patients with mutBRAF tumors.^21^ For these reasons, it is important to know the proper frequency, clinical characteristics, prognosis and treatment response in patients with MSI‐H and mut*BRAF* tumors in population‐based cohorts. The aim of this study was to analyze MSI‐status in relation to clinical and pathological variables, mut*BRAF* status and survival in a population‐based cohort of mCRC.

## 2 MATERIALS AND METHODS

### 2.1 Patient cohort

The study cohort is a prospective registration of non‐resectable mCRC patients referred to the oncology units of three university hospitals in Scandinavia (Odense University Hospital in Denmark, Uppsala University Hospital in Sweden and Haukeland University Hospital in Norway) between October 2003 and August 2006. Cases not referred (n = 49) were identified via the regional cancer registries. This cohort therefore includes all patients diagnosed with nonresectable mCRC in these three Nordic geographical regions. A total of 798 patients were included.^7^ The clinical data is from date of inclusion and was obtained from case report forms filled in by clinicians.

### 2.2 Tissue retrieval and tissue microarray generation

Paraffin‐embedded tissue blocks of the primary tumor or from a metastatic lesion were retrieved and corresponding hematoxylin‐eosin stained glass slides were examined. Tumor tissue from 462 cases (58%) was available for initial tissue microarray (TMA cohort) construction as described previously^7^ according to standards used in the Human Protein Atlas.^22^ DNA was extracted from the tissue cores using Recoverall Total Nucleic Acid Isolation (Ambion, Austin, TX). In the present study we supply additional analyses from patients without enough tumor material for TMA/DNA analysis (167 patients), called the immunohistochemistry (IHC) cohort. Totally 604 cases had tumor tissue available for analysis, as 25 cases failed due to technical reasons (Supplementary Figure S1).

### 2.3 Tumor analyses

*BRAF* and *KRAS* analyses of the TMA cohort had been done previously by pyrosequencing mutational analysis with 5% mutation signal as cut off, and the use of PCR primers for *KRAS* codon 12/13 and *BRAF* codon 600.^7^ MSI status for *BRAF‐*mutated patients in the TMA cohort had previously been obtained by DNA analysis using MSI Analysis System, version 1.2 (Promega, Madison, WI) with 6 ng genomic DNA.^7^

Immunohistochemistry (IHC) and image acquisition was performed according to standards used within the Human Protein Atlas.^23^  TMA sections 4 mm thick were subjected to heat‐induced antigen retrieval using PT module buffer 1 (pH 6, ThermoScientific) in a Decloaking Chamber (Biocare Medical), except for *BRAF* stained with special protocol HIER with TRIS‐EDTA at pH8. Automated IHC was performed using a LabVision Autostainer 480S (Thermo Fisher Scientific, Runcorn, UK). BRAF mutation was assessed with mouse antibody from Spring Bioscience, E19292, Clone VE1, diluted 1:50. MSH‐2 and MLH‐1 with mouse antibody from Becton Dickinson and Company (formerly PharMingen), Clone = G219‐1129 and G168‐15, diluted 1:200 and 1:100, respectively. PMS‐2 and MSH‐6 with rabbit antibody from Abcam plc, ab110638 clone = EPR3947 and ab92471 clone = EPR3945, diluted 1:75 and 1:125 respectively. IHC for V600E *BRAF* mutation was analyzed in both TMA and IHC cohorts. Cytoplasmic staining for *BRAF* mutation was qualitatively scored as positive (mutated) or negative (wildtype) in tumor cells. The results from the IHC and DNA analysis were compared and found inconsistent in seven cases. One of them had a V600R mutation discovered by sequencing and obviously not detected by the V600E IHC analysis. This patient was defined as mut*BRAF.* One patient turned out to be mut*BRAF* according to pyrosequencing, but wt*BRAF* according to IHC evaluation. This case had low amount of mutated DNA (8%), was mut*KRAS* and was therefore considered wt*BRAF*. The other five patients with inconsistent results were excluded from further analysis. A final *BRAF* status conclusion was made in 591 patients (Supplementary Figure S1 and Figure S2).

IHC for expression of MLH1, PMS2, MSH2, and MSH6 was performed for all patients included in the TMA cohort. Only MSH6 and PMS2 was analyzed in the additional IHC cohort due to limited amount of material in most cases. Nuclear fraction (NF) of the four MMR proteins were estimated. The samples were denoted as deficient DNA mismatch repair (dMMR) if complete loss of PMS2 or MSH6 staining. One patient had complete loss of MSH2 staining, but clearly positive staining for PMS2. This is an unexpected finding and the patient was therefore excluded from the final analysis. Results from IHC and DNA analysis were compared and merged, further referred to as MSI‐H or MSS, in 583 patients (Supplementary Figure S1).

### 2.4 Statistics

Group comparisons were performed using the exact chi‐square test for dichotomous or nominal variables and the log‐rank test for survival times. Multiple binary logistic regression was used for dichotomous outcome variables. Results are reported as odds ratios (ORs) and 95% confidence intervals (CIs). Overall survival time (OS) was the interval from the date of metastatic disease to the date of death or censored if the patient was alive on February 4, 2014. Progression‐free survival (PFS) was the interval from the date of first administration of chemotherapy to the date of progression (on CT scan) or death or censored if the patient was alive without progression on February 4, 2014. OS and PFS were analyzed using the Kaplan‐Meier method and Cox multiple regression. For the multivariate survival analyses we used Cox regression and backward stepwise selection of covariates to the final model. At the first step, we included all relevant covariates. These were prognostic variables for mCRC patients as recommended by Goey et al,^24^ only excluding the volume of liver involvement as this was not available. In addition, we included tumor grade, female sex and high alkaline phosphatase in blood samples as these variables are prognostic markers for survival. CEA >4 µg/L and high LDH were statistically significant when included in the multiple regression model, but were excluded from the analysis due to many missing values. From this model, we removed the variable with the largest *P*‐value >0.05. In the second step, we removed the covariate with the largest *P* > 0.05 among the remaining variables from the first step. The process continued until all the remaining variables were significant at level 0.05 and a final model was obtained. Results are reported as hazard ratios (HRs) and 95% CIs. All analyses were performed with the statistical program SPSS v22. All statistical tests were two‐tailed using significance level 5%.

## 3 RESULTS

### 3.1 Study population

In the 604 patients with sufficient morphological material of invasive adenocarcinoma for analyses (Supplementary Figure S1), the median age was 70 years and 209 patients (35%) were >75 years. In total 215 patients (36%) had PS >1. First‐line chemotherapy was given to 377 patients (62%, 75% below 75 years, and 26% above). Of those, 287 patients (76%) received combination chemotherapy, 28 patients (7%) received bevacizumab, and 27 patients (7%) received an EGFR‐inhibitor. Supplementary Table S1 illustrates follow‐up data on frequency of second‐ and third‐line treatment according to the different first‐line treatment given. Median OS (95% CI) was 11 months (9.6‐12.3) for all patients. For patients treated with first‐line chemotherapy, median OS and PFS were 17 months (15.0‐19.0) and 8 months (7.2, 8.4), respectively. At last follow‐up, 24 patients (3%) were alive.

### 3.2 MSI, *BRAF* status, and patient characteristics

**Figure 1:**
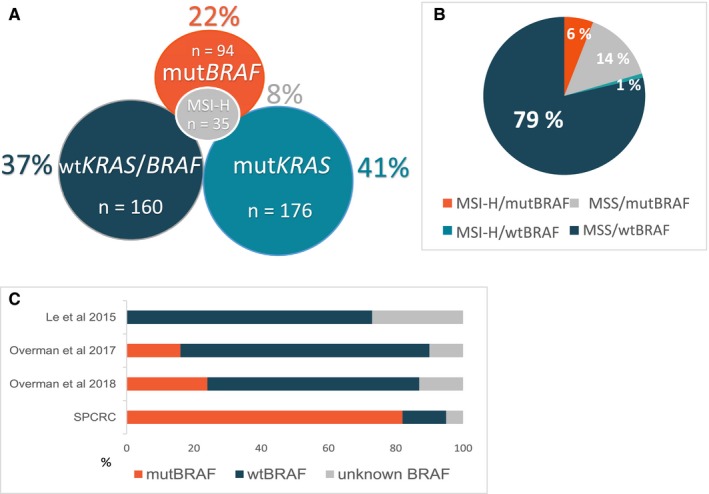
Mutation status in a population‐based Scandinavian cohort of metastatic colorectal cancer: (A) Venn diagram illustrating *KRAS*, *BRAF* and MSI status in primary tumors of patients in the TMA cohort with analysis available (n = 428), (B) Distribution of *BRAF*/MSI subgroups in patients with sufficient material for these analyses (n = 569), (C) Incidence (%) of *BRAF* mutations in MSI‐H tumors in the Scandinavian prospective colorectal cancer cohort (SPCRC) (n = 40) compared to recently published immunotherapy trials by Le et al 2015 (n = 21), Overman et al 2017 (n = 74) and Overman et al 2018 (n = 119)

Totally 40 (7%) of 583 evaluable tumors were MSI‐H. Tumors with MSI‐H status had more often mut*BRAF* compared to MSS tumors (87% vs 16%, *P* < 0.001), and consequently less often *KRAS* mutations (mut*KRAS*) (6% vs 44%, *P* < 0.001). Figure 1A illustrates *KRAS*, *BRAF*, and MSI status in the TMA cohort (n = 428). MSI‐H patients had less often liver and lung metastases, but more often lymph node metastases (Table 1). Female sex, right‐sided primaries, elderly patients, and grade 3‐4 tumors were more common in the MSI‐H group. Patients with MSI‐H tumors given first‐line palliative chemotherapy received less often second‐ and third‐line chemotherapy compared to MSS (30% vs 58%, *P* = 0.019 and 5% vs 27%, *P* = 0.033, respectively). In fully adjusted multiple logistic regression, right‐sided primaries, mut*BRAF*, and no lung metastases were significantly associated with MSI‐H status (Supplementary Table S2).

**Table 1:** Characteristics of a population‐based Scandinavian cohort of metastatic colorectal cancer patients with MSI status available (n = 583)

Characteristic n (%^a^)	All patients n = 583 (100%)	Missing *n*	MSI‐H n = 40 (7%)	MSS n = 543 (93%)	MSI‐H vs MSS *P*‐value
Age in years, *median*	70		75	70	0.157
Age >75 y, n (%)	200 (34)		19 (48)	181 (33)	0.084
Female, n (%)	281 (48)		28 (70)	253 (47)	0.005
PS ECOG >1, n (%)	207 (36)	1	18 (45)	189 (35)	0.231
ECOG 0	206 (35)		10 (25)	196 (36)	0.464
ECOG 1	169 (29)		12 (30)	157 (29)	
ECOG 2	117 (20)		11 (28)	106 (20)	
ECOG 3	90 (15)		7 (18)	83 (15)	
Right‐sided, n (%)	202 (35)	11	33 (85)	169 (32)	<0.001
Liver metastases, n (%)	378 (65)		13 (33)	365 (67)	<0.001
Liver only, n (%)	120 (21)		5 (13)	115 (21)	0.228
Lung metastases, n (%)	148 (27)		3 (8)	145 (27)	0.007
Lymph node metastases, n (%)	156 (27)		20 (50)	136 (25)	0.001
Peritoneal metastases, n (%)	108 (19)		6 (15)	102 (19)	0.676
> 1 metastatic site, n (%)	357 (61)		20(50)	337 (62)	0.178
Synchronous metastases, n (%)	332 (57)		23 (58)	309 (57)	1.000
Local relapse, n (%)	37 (6)		6 (15)	31 (6)	0.033
Comorbidity, n (%)	320 (56)	6	21 (54)	299 (56)	0.868
Weight loss >10%, n (%)	239 (45)	50	22 (60)	217 (44)	0.086
CEA >4 µg/L, n (%)	235 (78)	280	11 (69)	224 (78)	0.538
ALP high, n (%)	297 (57)	63	17 (57)	290 (57)	1.000
LDH high, n (%)	227 (48)	107	11 (37)	216 (48)	0.258
Primary tumor resected, n (%)	474 (81)		36 (90)	437 (81)	0.205
Tumor grade					<0.001
1‐2, n (%)	339 (79)	152	15 (43)	324 (82)	
3, n (%)	92 (21)		20 (57)	72 (18)	
*KRAS*					<0.001
Mutation, n (%)	177 (41)	151	2 (6)	175 (44)	
Wildtype, n (%)	255 (59)		34 (94)	221 (56)	
*BRAF*					<0.001
Mutation, n (%)	117 (21)	14	33 (87)	84 (16)	
Wildtype, n (%)	452 (79)		5 (13)	447 (84)	
Double wildtype, n (%)	160 (48)	249	3(60)	157 (48)	0.673
Curative surgery for metastases, n (%)	40 (7)	1	1 (3)	39 (7)	0.349
First‐line chemotherapy, n (%)	364 (62)		20 (50)	344 (63)	0.127
Combination chemotherapy, n (%)	278 (48)		14 (35)	264 (49)	0.103
Second‐line chemotherapy, n (%)	206 (36)	1	6 (15)	200 (37)	0.005
Third‐line chemotherapy, n (%)	92 (16)	1	1 (3)	91 (17)	0.022
Trial treatment, n (%)	131 (23)	1	7 (18)	124 (23)	0.448
BSC only, n (%)	216 (37)		19 (48)	197 (36)	0.176
Reason BSC, n (%)		36			0.049
Reduced general health	89 (50)		13 (87)	76 (46)	
Old age	27 (15)		0 (0)	27 (16)	
Comorbidity	16 (9)		2 (13)	14 (9)	
Patient declining treatment	26 (15)		0 (0)	26(16)	
Reduced liver function	4 (2)		0 (0)	4 (2)	
Other	18 (10)		0 (0)	18 (11)	

Abbreviations: ALP high, alkaline Phosphatase >105 U/L; BSC, best supportive care; CEA, carcinoembryonic antigen; Curative surgery, for metastatic disease; Double wildtype, both *BRAF* and *KRAS* wildtype; LDH high, Lactate Dehydrogenase above normal level according to age; Left sided, Site of colon cancer in descending colon, sigmoid and rectum; Metastases, at time of diagnosis of metastatic disease; MSI‐H, microsatellite instable high; MSS, microsatellite stable; PS ECOG, performance status score developed by Eastern Cooperative Oncology Group; *P*‐value, chi‐square test except for age (*t* test); Right sided, Site of colon cancer in ascending colon and transversum; Synchronous metastases, within 6 months after initial diagnose.

a Due to rounding not all percentages are 100 in total.

When analyzing all 591 patients with *BRAF* status available, the frequency of mut*BRAF* was 20% (120 of 591 patients) and MSI‐H was 7%. Tumors with mut*BRAF* were more often MSI‐H compared to wt*BRAF* (28% vs 1%, *P* < 0.001). We divided the patients into four groups according to MSI/*BRAF* status (Figure 1B, Supplementary Table S3). Elderly patients (>75 years) had more often MSI‐H/mut*BRAF* tumors compared to patients <75 years (9% vs 4%, *P* = 0.012). Patients with MSI‐H/mutBRAF tumors had also more often lymph node metastases and tumor grade 3, but less often liver metastases compared to the other groups. Patients with MSI‐H/wtBRAF tumors had more often liver metastasis as well as liver‐only metastasis compared to the other groups.

### 3.3 Overall and progression‐free survival

**Figure 2:**
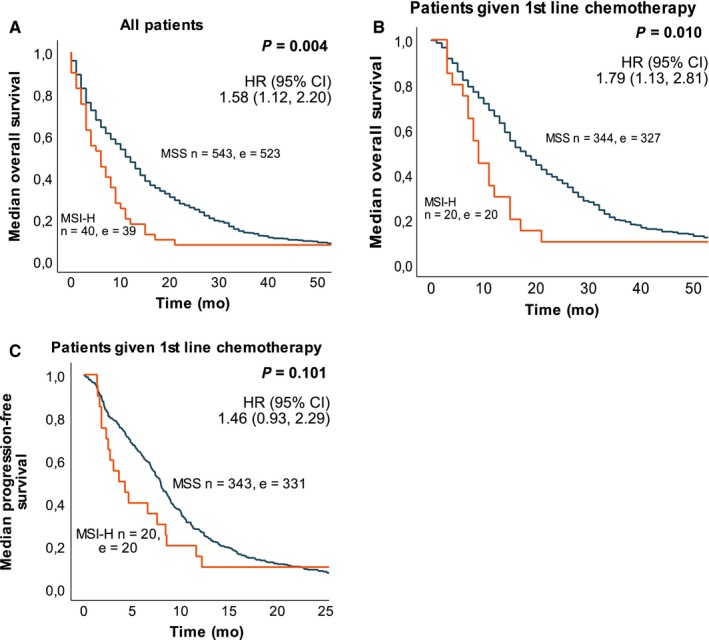
Survival in a population‐based Scandinavian cohort of patients with metastatic colorectal cancer according to MSI status. Kaplan‐Meier curves was calculated with log‐rank test for p‐value and univariate Cox regression for HR and 95% CI. A, Median overall survival for all patients with MSI status was 6 mo for patients with MSI‐H tumors and 11 mo for patients with MSS tumors. B, Median overall survival in patients given first‐line chemotherapy was 9 mo for patients with MSI‐H tumors and 18 mo for patients with MSS tumors. C, Median progression free survival in patients given first‐line chemotherapy was 4 mo for patients with MSI‐H tumors and 8 mo for patients with MSS tumors. n, number of patients; e, number of events, HR, Hazard Ratio, CI, confidence interval

**Figure 3:**
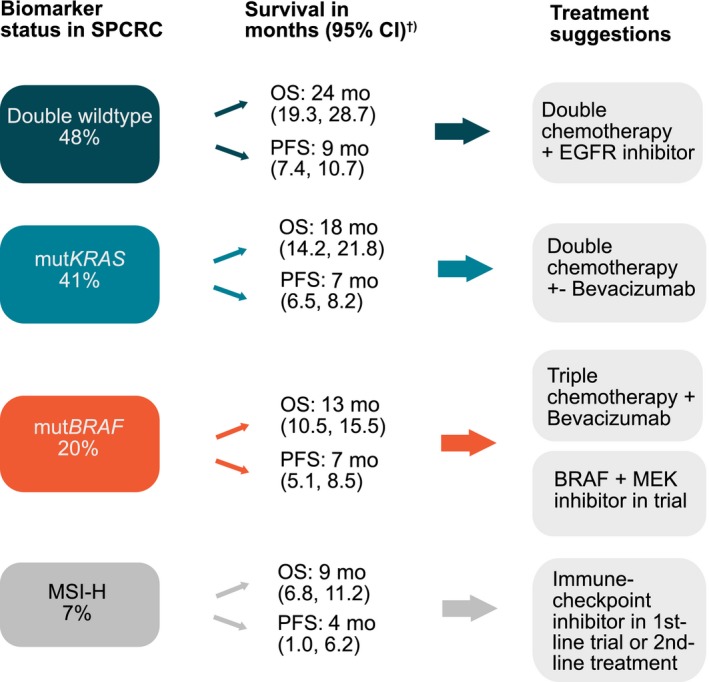
Frequency of molecular alterations and survival data after first‐line chemotherapy in a Scandinavian population‐based cohort of metastatic colorectal cancer (SPCRC) with suggestions on choice of treatment for the specific patient groups. Double wildtype, *BRAF* and *KRAS* wildtype; mut*BRAF*, *BRAF* mutation; mut*KRAS*, *KRAS* mutation; MSI‐H, microsatellite instability‐high; OS, median overall survival; PFS, median progression‐free survival; CI, confidence interval; †) after first‐line chemotherapy

Both median OS and PFS were shorter in patients with MSI‐H tumors (Figure 2, Table 2, Figure 3). Median OS was 6 months for patients with MSI‐H compared to 11 months for patients with MSS tumors (*P* = 0.004). Patients with mut*BRAF* tumors had a median OS of 7 months compared to 12 months with wt*BRAF* tumors (*P* < 0.001). Median OS in elderly patients was 4 versus 5 months for MSI‐H vs MSS cases (*P* = 0.024) and 3 versus 6 months (*P* < 0.001) for mut*BRAF* versus wt*BRAF* cases, respectively. For patients <75 years, median OS was 8 versus 15 months (*P* = 0.012) for MSI‐H vs MSS cases and 11 versus 16 months (*P* < 0.001) for mut*BRAF* vs wt*BRAF* patients, respectively. In the best supportive care group, median OS was 2 versus 3 months for MSI‐H versus MSS patients, respectively (*P* = 0.025). Among patients given first‐line chemotherapy, median OS was 9 versus 18 months for MSI‐H versus MSS cases (*P* = 0.010), and 13 versus 18 months for mut*BRAF* vs wt*BRAF* cases (*P* = 0.005). Median PFS after 1st‐line chemotherapy was 4 versus 8 months for MSI‐H versus MSS cases (*P* = 0.101) and 7 versus 8 months for mut*BRAF* versus wt*BRAF* cases (*P* = 0.125). For patients with response registered after first‐line chemotherapy with the MSI status (n = 321) and BRAF status (n = 328) available, the objective response rate (ORR) was 5% versus 44% for MSI‐H versus MSS cases (*P* = 0.002), and 37% versus 43% for mut*BRAF* versus wt*BRAF* cases (*P* = 0.609).

**Table 2:** Median (Med) overall survival and progression free survival (months) after different treatment regimens in a population‐based Scandinavian cohort of metastatic colorectal cancer patients (n = 583) according to MSI status (left side) and MSI and *BRAF*‐mutation status combined (right side)

n (%)	All patients 583 (100) Med (95% CI)	MSI‐H 40 (7) Med (95% CI)	MSS 543 (93) Med (95% CI)	*P*‐value	MSI‐H/mut*BRAF* 33 (6) Med (95% CI)	MSI‐H/wt*BRAF* 5 (1) Med (95% CI)	MSS/mut*BRAF* 84 (15) Med (95% CI)	MSS/wt*BRAF* 447 (79) Med (95% CI)	*P‐*value
Survival time									
OS									
All patients	11 (9.6, 12.4)	6 (2.9, 9.1)	11 (9.6, 12.4)	0.004	6 (2.6, 9.4)	2 (0.0, 4.1)	8 (3.9, 12.1)	12 (10.5, 13.5)	<0.001
n/e	583/562	40/39	543/523		33/33	5/5	84/83	447/428	
1st‐line chemotherapy	17 (14.9, 19.1)	9 (6.8, 11.2)	18 (15.8, 20.2)	0.010	11 (7.0, 15.0)	4 {4, 7}	14 (11.4, 16.6)	19 (16.4, 21.6)	0.001
n/e	364/347	20/20	344/327		17/17	2/2	51/50	287/271	
1st‐line combination chemotherapy	20 (17.3, 22.7)	8 (6.2, 9.8)	20 (17.3, 22.7)	0.015	8 (5.8, 10.2)	4 {4, 7}	14 (10.5, 17.5)	21 (18.1, 23.9)	<0.001
n/e	278/262	14/14	264/248		11/11	2/2	36/35	225/210	
Best supportive care only	3 (2.2, 3.8)	2 (0.3, 3.7)	3 (2.2, 3.8)	0.025	3 (1.1, 4.9)	1 {1, 1, 2}	1 (0.3, 1.7)	4 (3.2, 4.8)	0.001
n/e	216/215	19/19	197/196		16/16	3/3	33/33	158/157	
PFS									
1st‐line chemotherapy	8 (7.2, 8.4)	4 (1.0, 6.2)	8 (7.2, 8.6)	0.101	5 (0.6, 8.6)	2 {2, 2}	7 (5.5, 8.3)	8 (7.3, 8.7)	0.007
n/e	363/351	20/20	343/331		17/17	2/2	50/49	287/276	
1st‐line combination chemotherapy	8 (7.5, 8.8)	3 (0.2, 5.9)	8 (7.8, 9.0)	0.110	5 (0.8, 8.4)	2 {2, 2}	7 (6.7, 8.1)	9 (7.9, 9.2)	0.002
n/e	278/267	14/14	264/253		11/11	2/2	36/35	225/215	
2nd‐line chemotherapy	5 (3.7, 5.3)	4 (0.4, 7.9)	5 (3.7, 5.7)	0.578	3 (0.5, 4.9)	5 {5}	4 (1.3, 6.6)	5 (3.6, 5.9)	0.779
n/e	200/197	7/7	193/190		6/6	1/1	25/25	165/162	

Abbreviations: All patients, patients with MSI status available; CI, confidence interval; e, number of events; MSI‐H, microsatellite instable high; MSS, Microsatellite stable; mut*BRAF*, *BRAF* mutation; n, number of patients; OS, overall survival; PFS; progression free survival; *P*‐value, log‐rank test; wt*BRAF*, *BRAF* wildtype.

**Figure 4:**
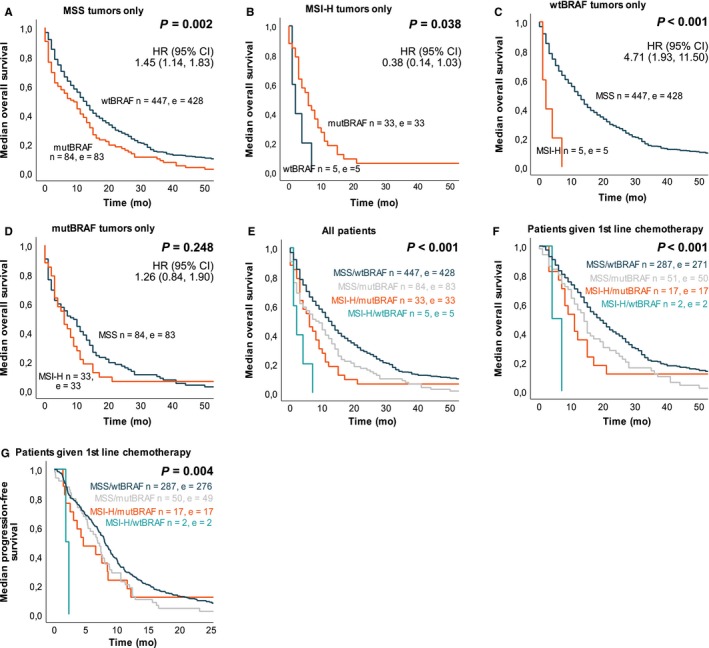
Survival in a population‐based Scandinavian cohort of metastatic colorectal cancer patients according to MSI and *BRAF* status. Kaplan‐Meier curves was calculated with log‐rank test for p‐value and univariate Cox regression for HR and 95% CI. A, Median overall survival for patients with MSS tumors was 8 mo if mut*BRAF* and 12 mo if wt*BRAF*. B, Median overall survival for patients with MSI‐H tumors was 6 mo if mut*BRAF* and 2 mo if wt*BRAF*. C, Median overall survival for patients with *BRAF*‐wildtype tumors was 2 mo if MSI‐H and 12 mo if MSS. D, Median overall survival for patients with *BRAF‐*mutated tumors was 6 mo if MSI‐H and 8 mo if MSS. E, Median overall survival for all patients in subgroups of MSI and *BRAF* status. F, Median overall survival for patients given first‐line chemotherapy. G, Median progression free survival for patients given first‐line chemotherapy. n, number of patients; e, number of events

In log‐rank survival analyses, the negative prognostic potential of MSI‐H was only statistically significant in wt*BRAF* tumors and the negative prognostic potential of mut*BRAF* was only seen in MSS tumors (Figure 4A‐D, supplementary Figure S3). The test of interaction between these two variables was significant (*P* = 0.010), also when adjusting for other prognostic variables (*P* = 0.037, Table 3). When dividing patients into four subgroups according to *BRAF* and MSI status, median OS and PFS after first‐line chemotherapy was statistically significantly different (Figure 4E‐G, Table 2). Patients with MSI‐H/wtBRAF had the worst prognosis compared to the other groups with only 2 months median OS and PFS, but we had only five patients with this molecular tumor characteristic. The negative prognostic value of MSI‐H status was seen regardless of *KRAS* status, and in patients with wild‐type *KRAS* and wt*BRAF* (double wildtype) tumors, but we had very few patients in some of the subgroups (Supplementary Table S4).

**Table 3:** Results from multiple Cox regression of overall survival and progression free survival in a population‐based Scandinavian cohort of metastatic colorectal cancer patients (n = 798) diagnosed from October 2003 to August 2006 and followed until 4 February 2014

	Overall survival all patients^a^) (n = 360, e = 343)			Overall survival in patients given 1st‐line chemotherapy (n = 248, e = 233)			Progression free survival after 1st‐line chemotherapy (n = 247, e = 237)		
Variable	HR	95% CI	*P*‐value	HR	95% CI	p‐value	HR	95% CI	*P*‐value
Female	0.85	0.68‐1.06	0.156	0.77	0.59‐1.02	0.063	0.97	0.74‐1.26	0.805
Age >75 y	1.05	0.75‐1.48	0.757	1.39	0.89‐2.16	0.147	1.54	0.99‐2.38	0.055
PS ECOG > 1	1.86	1.42‐2.44	<0.001	2.17	1.50‐3.13	<0.001	2.05	1.42‐2.96	<0.001
Right‐sided tumor	1.11	0.86‐1.43	0.407	0.96	0.71‐1.31	0.815	0.79	0.58‐1.08	0.135
Tumor grade 3	1.81	1.34‐2.45	<0.001	1.76	1.22‐2.55	0.003	1.65	1.15‐2.37	0.007
Primary tumor resected	1.04	0.60‐1.83	0.879	0.80	0.43‐1.49	0.480	1.48	0.83‐2.63	0.185
Synchronous metastases	0.70	0.55‐0.88	0.002	0.78	0.59‐1.04	0.087	0.77	0.58‐1.02	0.066
> 1 organ metastases	1.55	1.14‐2.10	0.005	1.79	1.22‐2.63	0.003	1.48	1.01‐2.16	0.044
Liver only	1.44	1.00‐2.08	0.051	1.44	0.92‐2.26	0.113	1.34	0.86‐2.09	0.198
Curative metastasis surgery	0.29	0.18‐0.47	<0.001	0.33	0.20‐0.55	<0.001	0.38	0.24‐0.62	<0.001
ALP high	2.00	1.57‐2.54	<0.001	1.95	1.45‐2.63	<0.001	1.59	1.48‐2.13	0.002
First‐line chemotherapy	0.38	0.27‐0.54	<0.001			ni			ni
MSI‐H	1.42	0.86‐2.37	0.174	2.34	1.18‐4.64	0.015	2.13	1.08‐4.18	0.028
*BRAF* mutation	1.86	1.29‐2.69	0.001	1.94	1.23‐3.05	0.004	1.62	1.04‐2.53	0.034
*KRAS* mutation	1.22	0.94‐1.57	0.135	1.39	1.02‐1.90	0.038	1.52	1.12‐2.08	0.008
Interactions^b^									
MSI‐H effect in wt*BRAF*	4.46	1.83‐10.86	0.001						
MSI‐H effect in mut*BRAF*	1.20	0.80‐1.80	0.386						
mut*BRAF*‐effect in MSS	1.44	1.14‐1.83	0.002						
mut*BRAF*‐effect in MSI‐H	0.40	0.16‐1.04	0.059						

Abbreviations: ALP high, Alkaline Phosphatase >105 U/L; CI, confidence interval; e, number of events; HR, hazard ratio; MSI‐H, microsatellite instable high; MSS, microsatellite stable; mut*BRAF*, *BRAF* mutated; n, number of patients; ni, not included; PS ECOG, performance status score developed by Eastern Cooperative Oncology Group; *P*‐value, from likelihood ratio test; wt*BRAF*, *BRAF* wildtype.

a CEA >4 and LDH high was also statistically significant when included in the multiple regression model, but were excluded from the analysis due to many missing values.

b Testing the hypothesized interaction between MSI and *BRAF* showed significantly higher effect of MSI‐H among those with wt*BRAF* (HR = 4.46) than in those with mut*BRAF* (HR = 1.20) tumors and higher effect of mut*BRAF* among those with MSS (HR = 1.44) compared to MSI‐H (HR = 0.40) tumors (interaction HR = 0.28, 95% CI: 0.11‐0.74, P = 0.010), after adjusting for all other covariates it was still statistically significant (interaction HR = 0.20, 95% CI: 0.94‐0.91, *P* = 0.037).

In multiple Cox regression analyses, including known clinical prognostic factors for mCRC survival, mut*BRAF* was the only molecular tumor marker significantly associated with reduced OS. For patients who received first‐line chemotherapy mut*BRAF*, mut*KRAS* and MSI‐H were all significantly associated with shorter OS and PFS (Table 3).

## 4 DISCUSSION

This is, as far as we know, the largest population‐based study reporting on MSI and *BRAF* status and its effect on treatment and survival in mCRC. The general poor survival in our cohort is comparable to that seen in Scandinavian cancer registries (10 months median OS) (8) and the American SEER database (1 year survival rate 47%) 25 during the same time period, reflecting our real‐world cohort of patients and not poor treatment, as patients receiving combination chemotherapy had the same OS as in clinical trials, including patients during the same time period. We report 7% MSI‐H tumors, almost twice as high as most previous reports of mCRC.^1^, ^2^, ^3^, ^4^, ^5^ We believe this is due to the unselected nature of the cohort with many elderly patients, patients with poor PS, never included in clinical trials, and many mut*BRAF* cases. Tran et al observed 8% MSI‐H in a trial population group and 13% in a general population group, also showing higher presence of MSI‐H in patients outside clinical trials.^6^ A recently published study on genomic profiling of 8887 mCRC patients reported 7% MSI‐H cases,^26^ in accordance with our result. In this study, we also confirm our previously published result showing a much higher frequency of mut*BRAF* tumors (20%) compared to previous studies.^7^ The same frequency is reported in a recent Nordic phase II trial of elderly vulnerable patients with mCRC.^27^ The relatively higher frequency of MSI‐H and mut*BRAF* in the general mCRC population implicates that more patients than previously expected could benefit from immunotherapy, or being candidate for adding *BRAF*‐inhibitor combinations to standard treatment (Figure 3). This stresses the importance of MMR and *BRAF* testing in all mCRC patients. Figure 3 illustrates the distribution of the tumor molecular alterations, their survival and possible treatment options.

In primary, non‐metastatic CRC, MSI‐H is known to be a good prognostic factor.^28^, ^29^ In mCRC, on the other hand, these patients belong to the poor prognostic group, in accordance with previous studies.^2^, ^3^, ^5^, ^6^, ^14^ MSI‐H and mut*BRAF* were clearly associated, and in multivariate analysis both tumor markers were independent poor prognostic predictors for OS and PFS in patients treated with first‐line chemotherapy. In subgroup analysis, we found that the negative impact of MSI‐H on survival only reached statistical significance in wt*BRAF* patients, and the negative impact of mut*BRAF* on survival was only seen in MSS patients. Previous studies on this matter have shown contradictory results.^3^, ^4^, ^5^, ^6^, ^16^, ^30^ None of the other studies are entirely population based, and a limitation of all studies, including our own, is the limited number of patients in some subgroups. In our cohort, patients with MSI‐H/wt*BRAF* tumors had the worst prognosis, in accordance with the randomized COIN trial.^3^ A recent study reports poor prognosis in mCRC patients with ALK, ROS1, and NTRK rearrangements in tumor, and these cases were associated with MSI‐H (48%) and were almost exclusively wt*BRAF* (96%).^31^ This might explain the particularly poor prognosis we see in this subpopulation, but the very limited number of patients in this group precludes firm conclusions and the results need verification in the larger studies.

Our cohort of MSI‐H patients had substantially less benefit from chemotherapy compared to MSS patients, and very few made it to the second‐ and third‐line of treatment. Recent third‐line immunotherapy trials in MSI‐H mCRC patients have shown ORR 31% (nivolumab monotherapy) and 55% (dual checkpoint inhibition), with median PFS and OS not reached at 12 months.^19^, ^20^ These impressive results are in great contrast to the treatment benefit (ORR 5%, PFS 4 months) and survival (OS 9 months) seen in our unselected MSI‐H patients treated with first‐line chemotherapy. However, the patient populations differ greatly and there are probably several reasons for this vast difference in prognosis. More than 45% of our MSI‐H patients had PS > 1, while immunotherapy trials only include patients with PS 0‐1. Most MSI‐H patients in our cohort were also mut*BRAF* (87%) (Figure 1C), markedly in contrast to the immunotherapy trials, where only 0%, 16%, and 24% were mut*BRAF* 18, 19, 20 (Figure 1D‐F). Other population‐based cohorts of mCRC have shown different frequencies of mut*BRAF* in MSI‐H tumors, ranging from 25%‐60%, but with limited number of patients.^4^, ^6^, ^16^, ^29^, ^32^ Patients with Lynch syndrome tumors are essentially MSI‐H/wt*BRAF* and often diagnosed at a younger age, which could explain the high frequency of these patients in clinical studies. In our study, we found only five cases with this molecular feature. MSI‐H/mut*BRAF* tumors develop in the serrated pathway and belong to the consensus molecular subtype 1 classification of CRC, associated with poor survival after relapse.^33^ In the two immunotherapy trials including patients with mut*BRAF* tumors, the response rate and survival did not significantly differ according to *BRAF* status, but the numbers were limited. Additional data on immunotherapy in the non‐Lynch group in a general patient population is warranted, to further evaluate if the benefit of immunotherapy in MSI‐H patients may vary according to *BRAF* status.

In our study, MSI‐H/mut*BRAF* cases were more often seen in elderly patients (>75 years), and median age of MSI‐H cases was 75 years, in great contrast to the recent third‐line immunotherapy trials (46‐58 years).^18^, ^19^, ^20^ MSI‐H status was less important for survival in elderly patients, but this might in part reflect the low treatment frequency in this subgroup. Elderly cancer patients in general receive less palliative chemotherapy and treatment recommendations for the elderly are uncertain as they usually are not included in clinical trials.^8^ In a recent retrospective study, elderly patients (>62 years) with malignant melanoma had a better response to anti‐PD‐1 therapy compared to younger patients,^34^ believed to be due to decreased intertumoral Tregs and increased CD8^+^:Treg ratio in the elderly patients. Considering the high age of most mCRC patients with MSI‐H tumors, future studies on immunotherapy in elderly patients are important.

The marked difference between our MSI‐H patients and third‐line immunotherapy trials reported so far should be taken into consideration when transferring results from these studies to the general population. There is reason to believe that future immunotherapy trials in first‐line may recruit a more heterogeneous MSI‐H population, with for instance more aggressive disease and more mut*BRAF* cases.

At present, the use of immune‐checkpoint inhibitors is recommend as second‐line treatment for MSI‐H mCRC patients.^21^ Our data, however, show that a substantial number of MSI‐H patients never get to second‐line treatment and the benefit of first‐line chemotherapy is very limited. Both these factors indicate that checkpoint inhibitors should probably be given as first‐line treatment and such studies are ongoing. A recent abstract from the Checkmate 142 trial with first‐line dual checkpoint inhibition in 45 patients reported 60% ORR, in line with third‐line immunotherapy trials.^35^ The very poor response rate in our cohort of 5% in MSI‐H compared to 40% in MMS is of particular concern if the patient has a potentially resectable disease, and first‐line treatment with checkpoint inhibitors should be considered in such cases.

## 5 LIMITATIONS OF STUDY

This is a prospectively collected study; the analyses, however, were done retrospectively. The patients were treated more than 10 years ago, and although the treatment options for mCRC have not changed much in the past decade, we possibly treat more patients with more intensive/combination regimens as well as metastasectomy, resulting in improved survival. Our study is population‐based and therefore includes more patients with older age, worse PS, comorbidity and less treatment compared to clinical trials, however, patients without available or sufficient tumor tissue for analysis could not be included and these patients have a particularly poor prognosis.^7^ Despite this being the largest population‐based study reporting on MSI and BRAF status and its effect on survival in mCRC, the number of patients in some of the MSI/*BRAF* subgroups was still limited.

## 6 CONCLUSIONS

In a population‐based cohort of mCRC patients, MSI‐H and mut*BRAF* were more common than previously reported, and consequently more patients could benefit from immunotherapy and *BRAF*‐inhibitor treatments. Our unselected cohort of MSI‐H patients differed considerably from patients included in recent immunotherapy trials as they were older, had worse PS and most of them also had mut*BRAF* tumor (non‐Lynch). Further studies are needed to evaluate the effect of immunotherapy in these subgroups of patients. Patients with MSI‐H tumors had worse survival, very poor response rate and few received second‐line treatment, indicating that these patients should probably be considered for immunotherapy as the first‐line treatment.

## CONFLICT OF INTEREST

The authors of this manuscript declare no potential conflicts of interest.

## AUTHOR CONTRIBUTIONS

HS: conceptualization, data curation, methodology, project administration, supervision, writing—original draft and writing—review and editing. PP: conceptualization, data curation, methodology, project administration, supervision and writing—review and editing. BG: conceptualization, data curation, methodology, project administration, supervision, resources, writing—original draft and writing—review and editing. AD: investigation, methodology, validation and writing—review and editing. FP: investigation, methodology, resources, validation and writing—review and editing. PHE: investigation, methodology, validation and writing—review and editing. AM: investigation, methodology, validation and writing—review and editing. MS: investigation, methodology, validation and writing—review and editing. KA: formal analysis, visualization, writing—original draft and writing—review and editing. GEE: formal analysis, methodology and writing—review and editing.

## ETHICS APPROVAL AND CONSENT TO PARTICIPATE

This study was performed in accordance with the Declaration of Helsinki. Written informed consent was obtained from all patients seen at the clinics. The study, including molecular classification of all tumors in the three regions, was approved by the regional ethical committees in Norway (Regional Committee for Medical and Health Research Ethics—REC West), Sweden (Regional Ethical Committee Uppsala) and Denmark (The Regional Scientific Ethical Committees for Southern Denmark).

## DATA AVAILABILITY STATEMENT

The data that support the findings of this study are available from the corresponding author upon reasonable request.
