# Supplementary material for: Consequences of a high incidence of microsatellite instability and BRAF‐mutated tumors: A population‐based cohort of metastatic colorectal cancer patients
Source: Cancer Med. 2019 May 9;8(7):3623–35. doi: 10.1002/cam4.2205 (PMC6601706; doi:10.1002/cam4.2205)

Supplementary material

Table S1. Frequency of different treatment regimens given in 1st line treatment and its impact on frequency of further 2nd and 3rd line treatment in a population-based cohort of 377 mCRC patients with sufficient morphological material of invasive adenocarcinoma for analyses

1st-line treatment regimen given	n (%)	n (%) received 2nd-line†	n (%) received 3rd-line†
Combination chemotherapy	287 (76%)	184 (64%)	81 (28%)
FLIRI	71 (19%)	51 (72%)	24 (34%)
FLOX	213 (57%)	133 (63%)	57 (27%)
FOLFOXIRI	3 (1%)	0	0
FLV monotherapy	82 (22%)	25 (31%)	13 (16%)
Irinotecan monotherapy	8 (2%)	4 (50%)	1 (13%)

Abbreviations: FLIRI: 5-fluorouracil, Folinic acid, Irinotecan; FLOX: 5-fluorouracil, Folinic acid, Oxaliplatin; FOLFOXIRI; 5-fluorouracil, Folinic acid, Oxaliplatin, Irinotecan; FLV: 5-fluorouracil, Folinic acid †) of patients that received 1st -line chemotherapy

Table S2. Results from multiple logistic regression of microsatellite-instable high status in a population based Scandinavian cohort of metastatic colorectal cancer patients (n = 583)

	Unadjusted models				Fully adjusted model (n = 412)			Simplified model †) (n = 559)		
**Predictor variables**	**n**	**OR**	**95 % CI**	**p-value**	**OR**	**95 % CI**	**p-value**	**OR**	**95 % CI**	**p-value**
Female	583	2.68	(1.33, 5.37)	0.004	2.01	(0.76, 5.31)	0.152			
Right-sided tumor	572	11.85	(4.87, 28.81)	< 0.001	5.06	(1.51, 16.90)	0.004	6.78	(2.45, 18.78)	< 0.001
Elderly (> 75 years)	583	1.81	(0.95, 3.45)	0.075	2.08	(0.82, 5.26)	0.120			
KRAS mutation	432	0.07	(0.02, 0.31)	< 0.001	0.41	(0.07, 2.45)	0.312			
BRAF mutation	569	35.12	(13.33, 92.56)	< 0.001	8.26	(2.26, 30.17)	< 0.001	17.64	(6.44, 48.30)	< 0.001
Tumor grade 3	431	6.00	(2.93, 12.28)	< 0.001	2.14	(0.81, 5.65)	0.127			
Liver metastases	583	0.24	(0.12, 0.47)	< 0.001	0.47	(0.18, 1.22)	0.121	0.40	(0.18, 0.90)	0.025
Lung metastases	583	0.22	(0.07, 0.73)	0.003	0.22	(0.04, 1.14)	0.040			
Peritoneal metastases	583	0.76	(0.31, 1.87)	0.542	0.44	(0.14, 1.39)	0.148			
Lymph node metastases	583	2.99	(1.56, 5.73)	0.001	1.35	(0.49, 3.68)	0.562			
Local relapse	583	2.92	(1.14, 7.47)	0.043	2.03	(0.34, 12.00)	0.438			

Abbreviations: Right sided tumor: Site of colon cancer in ascending colon and transversum; Tumor grade 3-4: low and undifferentiated; n: number of patients; OR: Odds ratio; CI: confidence interval; p-value: likelihood ratio test. †) from backward stepwise selection at nominal significance level 0.05.

Table S3. Patient characteristics in a population based Scandinavian cohort of 569 patients with metastatic colorectal cancer according to subgroups of *BRAF* and MSI status

**Characteristics *n (%†)***	**All patients ‡)**	**Missing**	**MSI-H/ mut*BRAF***	**MSS/ mut*BRAF***	**MSI-H/ wt*BRAF***	**MSS/ wt*BRAF***	**p-value**
**Total number,** *n (% of total* number)	569		33 (6)	84 (15)	5 (1)	447 (79)	
**Age in years,** median	70		76	68	70	69	
**Age > 75 years,** *n (%)*	194 (34)		18 (55)	26 (31)	1 (20)	149 (33)	0. 063
**Female,** *n (%)*	274 (48)		23 (70)	51 (61)	4 (80)	196 (44)	< 0.001
**PS ECOG >1,** *n (%)*	201 (35)	1	15 (46)	34 (41)	2 (40)	150 (34)	0.354
**Right-sided,** *n (%)*	198 (35)	10	28 (88)	45 (54)	4 (80)	121 (28)	< 0.001
**Liver metastases,** *n (%)*	370 (65)		9 (27)	46 (55)	4 (80)	311 (70)	< 0.001
**Liver only,** *n (%)*	119 (21)		3 (9)	8(10)	2 (40)	106 (24)	0.007
**Lung metastases,** *n (%)*	146 (26)		3 (9)	23 (27)	0	120 (27)	0.071
**Lymph node metastases,** *n (%)*	151 (27)		17 (52)	29 (35)	2 (40)	103 (23)	0.001
**Peritoneal metastases,** *n (%)*	106 (19)		5 (15)	22 (26)	1 (20)	78 (17)	0.251
**>1 metastatic site,** *n (%)*	348 (61)		17 (52)	53 (63)	2 (40)	276 (62)	0.492
**Synchronous metastases,** *n (%)*	325 (57)		21 (64)	53 (63)	2 (40)	249 (56)	0.449
**Comorbidity,** *n (%)*	314 (56)	6	20 (61)	45 (56)	1 (25)	248 (56)	0.617
**Weight loss >10%,** *n (%)*	232 (45)	49	19 (59)	38 (51)	2 (67)	173(42)	0.129
**CEA >4 µg/L,** *n (%)*	230 (78)	273	9 (69)	26 (74)	1 (50)	194 (79)	0.594
**ALP high,** *n (%)*	291 (57)	62	15 (56)	32 (44)	1 (50)	243 (60)	0.097
**LDH high,** *n (%)*	223 (48)	104	9 (33)	26 (40)	2 (100)	186 (50)	0.059
**Primary tumor resected,** *n (%)*	464 (82)		29 (88)	71 (85)	5 (100)	359 (80)	0.377
**Tumor grade 1-2,** *n (%)*	334 (78)	143	10 (35)	41 (66)	4 (80)	279 (85)	< 0.001
**3,** *n (%)*	92 (22)		19 (66)	21 (34)	1 (20)	51 (16)	
***KRAS* mutation,** *n (%)*	176 (41)	141	0	2 (3)	2 (40)	172 (52)	< 0.001
**wildtype,** *n (%)*	252 (59)		30 (100)	62 (97)	3 (60)	157 (48)	
**Curative surgery for metastases,** *n (%)*	38 (7)	1	0	1 (1)	0	37 (8)	0.058
**1st line chemotherapy,** *n (%)*	357 (63)		17 (52)	51 (61)	2 (40)	287 (64)	0.340
**2nd line chemotherapy,** *n (%)*	203 (36)	1	6 (18)	27 (32)	0	170 (38)	0.030
**3rd line chemotherapy,** *n (%)*	92 (16)	1	1 (3)	8 (10)	0	83 (19)	0.021
**Trial treatment,** *n (%)*	130 (23)	1	6 (18)	20 (24)	1 (20)	103 (23)	0.912
**BSC only,** *n (%)*	210 (37)		16 (49)	33 (39)	3 (60)	158 (35)	0.294

Abbreviations: MSI-H: microsatellite instable high; MSS: microsatellite stable; mutBRAF: BRAF mutation; wtBRAF: BRAF wildtype; PS ECOG: performance status score developed by Eastern Cooperative Oncology Group; Right sided tumor: Site of colon cancer in ascending colon and transversum; Metastases: at time of diagnosis of metastatic disease; Synchronous metastases: within 6 months after initial diagnose; CEA: Carcinoembryonic Antigen; ALP high: Alkaline Phosphatase > 105 U/L; LDH high: Lactate Dehydrogenase above normal level according to age; Tumor grade 1-2: well and medium differentiated; Tumor grade 3-4: low and undifferentiated; Curative surgery: for metastatic disease; BSC: Best supportive care; p-value: chi-square test †) Due to rounding not all percentages are 100 in total. ‡) all patients with MSI and BRAF status available

Table S4. Median (Med) overall survival (OS) in a population based Scandinavian cohort of metastatic colorectal cancer patients (n = 432) in subgroups of microsatellite instability status and *KRAS*-mutation status

	**n/e**	**Med OS (95 % CI)**	**p-value**
MSI-H / mut*KRAS*	2/2	1 {0.0, 0.0}	< 0.001
MSI-H / wt*KRAS*	34/33	5 (2.6, 7.4)	
MSS/ mut*KRAS*	175/168	13 (10.8, 15.2)	
MSS/ wt*KRAS*	221/211	13 (10.6, 15.4)	
MSI-H/doublewt	3/3	4 (0.8, 4.2)	0.006
MSS/doublewt	157/148	15 (10.5, 19.5)	

Abbreviations: MSI-H: microsatellite instable high; MSS: Microsatellite stable; mutKRAS: KRAS mutation; wtKRAS: KRAS wildtype; doublewt: *KRAS* and *BRAF* wildtype; n: number of patients; e: number of events; CI: confidence interval; p-value: log-rank test

Figure S1. Collection of tumor blocks and tissue micro array (TMA), Microsatellite instability status (MSI) and *BRAF* mutation availability with immunohistochemistry analysis and/or DNA sequencing in a population based Scandinavian cohort of 798 metastatic colorectal cancer (mCRC) patients

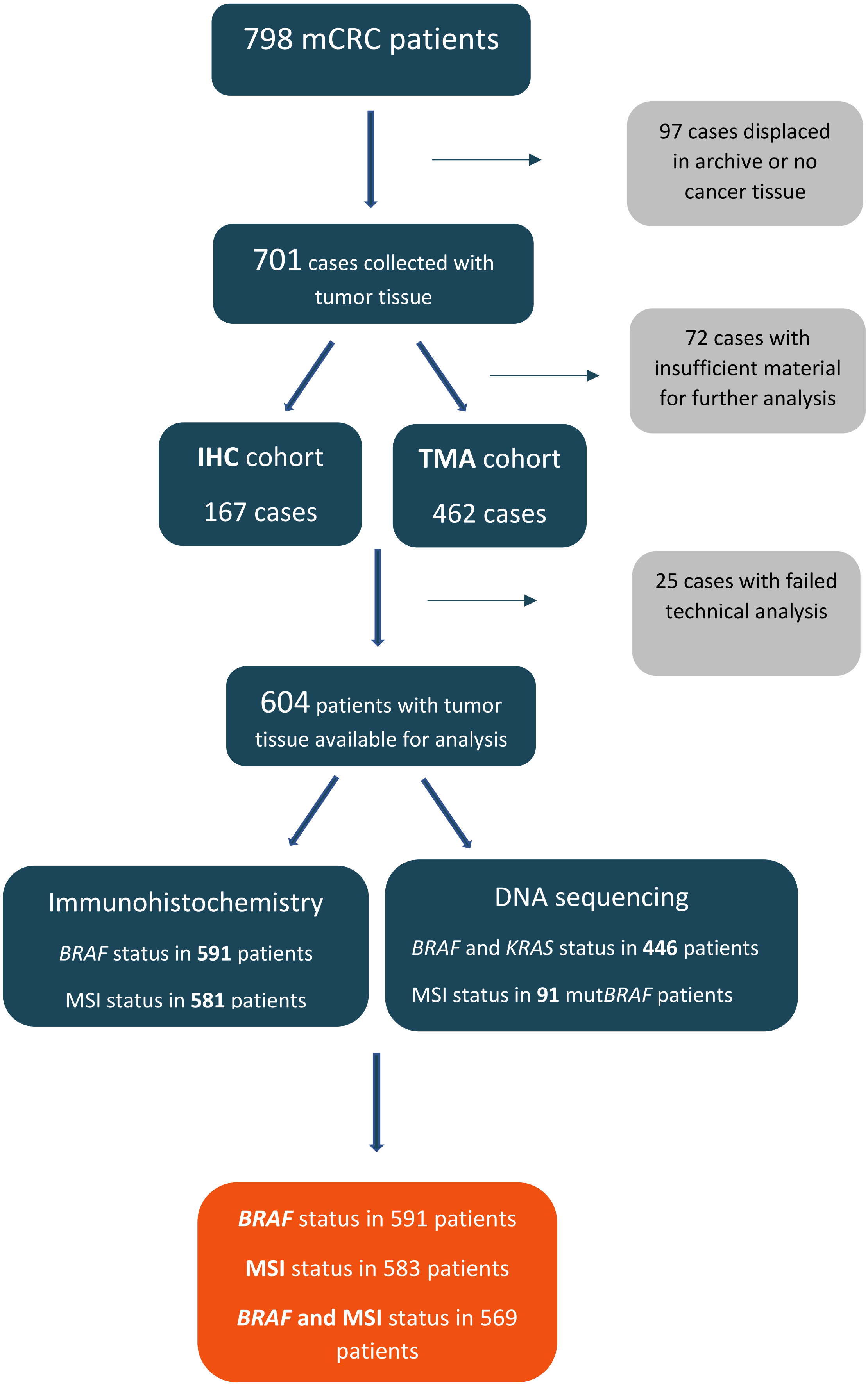

Figure S2. Final decision on BRAF status (right column) in seven cases with inconsistent result from DNA pyrosequencing with PCR primers for codon 600 (left column) and immunohistochemistry staining for V600E (middle column).

Abbreviations: mut: mutation; wt: wildtype

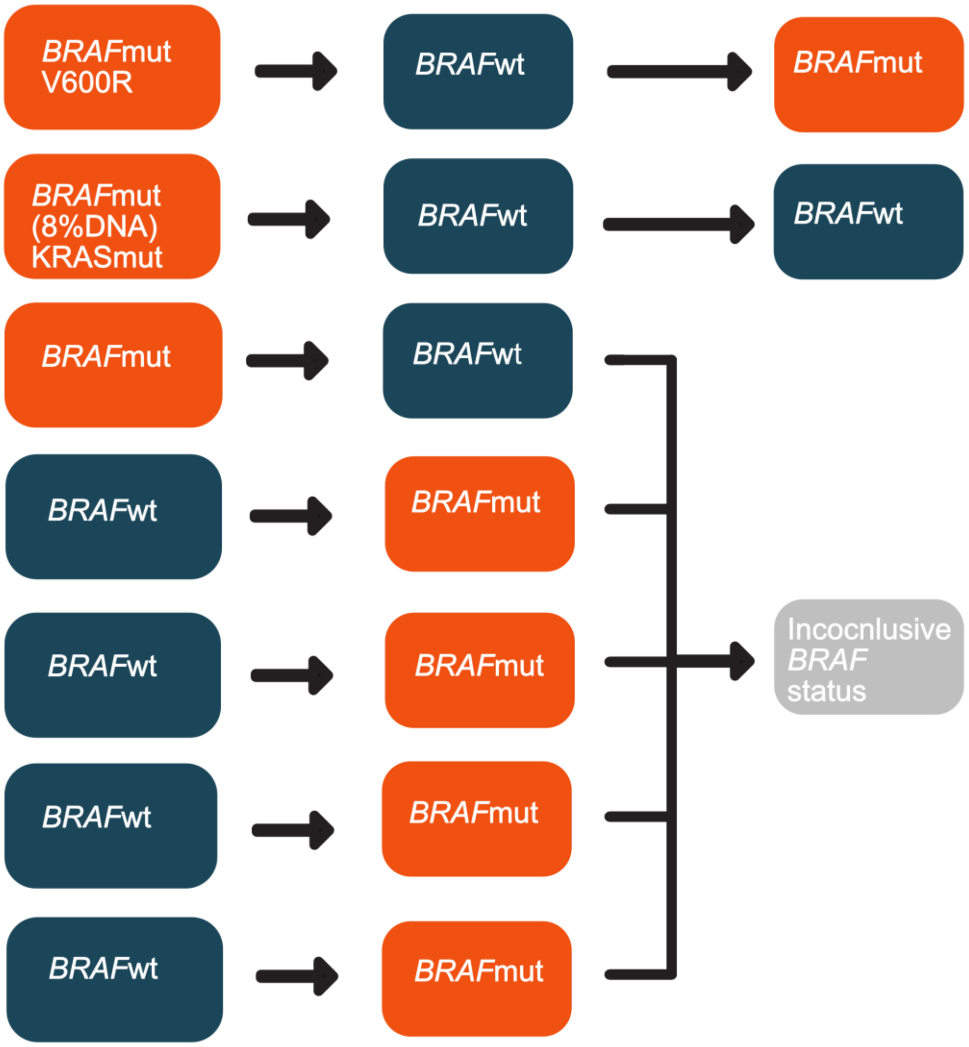

Figure S3. Flow chart of median overall survival in months with 95% confidence interval according to different treatments given in a population based Scandinavian cohort of metastatic colorectal cancer (mCRC) with *BRAF* and Microsatellite instability status available for analysis

Abbreviations: mCRC: metastatic colorectal cancer; BSC: Best supportive care; MSI-H: microsatellite instable high; MSS: microsatellite stable; mut*BRAF*: *BRAF* mutated; wt*BRAF*: *BRAF* wildtype; values in italics: p-value not significant (p>0.05)

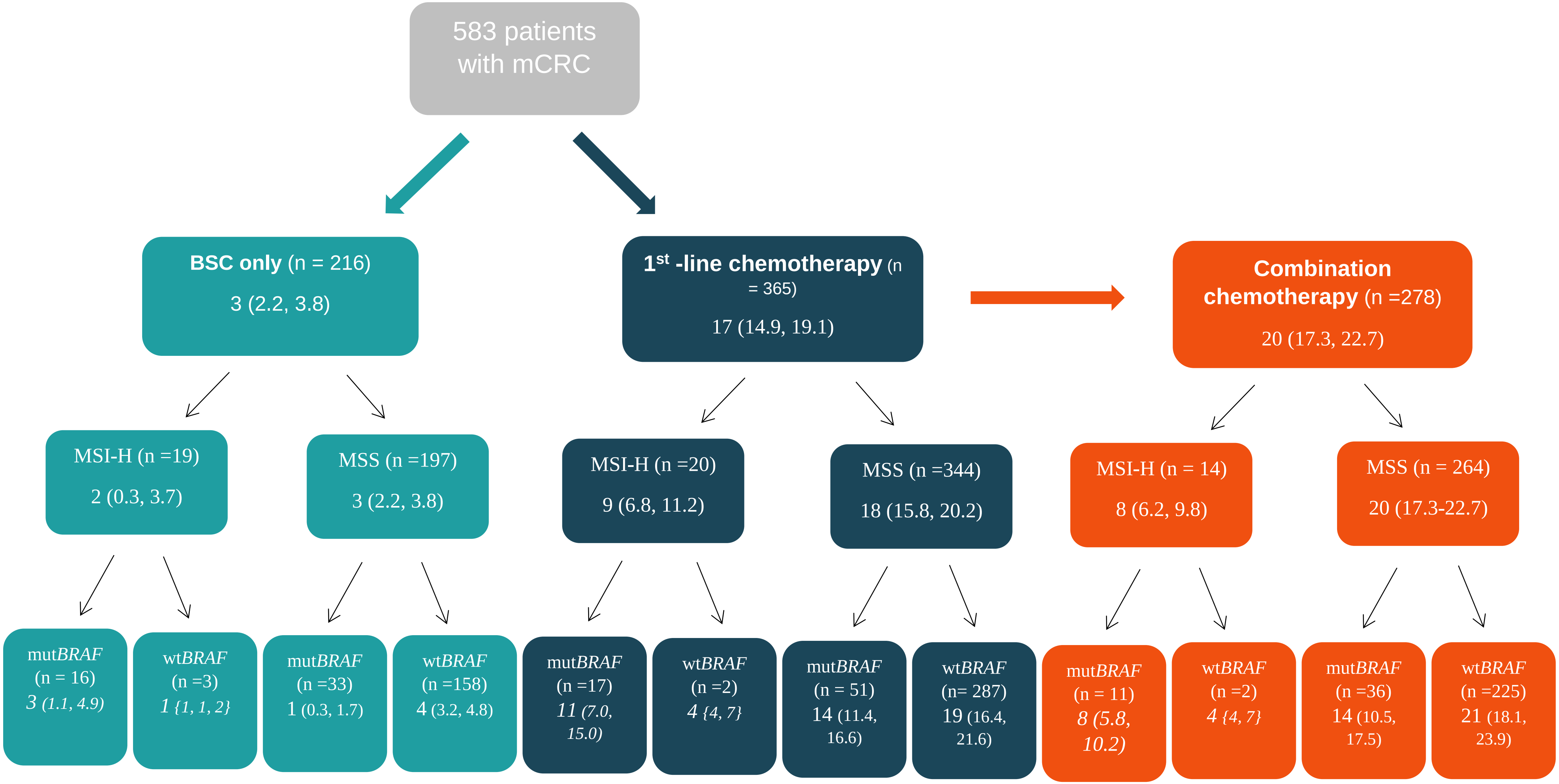

Supplement: Supplementary file 1 [file CAM4-8-3623-s001.pdf]
